# An Online Repository for Pre-Clinical Imaging Protocols (PIPs)

**DOI:** 10.3390/tomography9020060

**Published:** 2023-03-27

**Authors:** Seth T. Gammon, Allison S. Cohen, Adrienne L. Lehnert, Daniel C. Sullivan, Dariya Malyarenko, Henry Charles Manning, David A. Hormuth, Heike E. Daldrup-Link, Hongyu An, James D. Quirk, Kooresh Shoghi, Mark David Pagel, Paul E. Kinahan, Robert S. Miyaoka, A. McGarry Houghton, Michael T. Lewis, Peder Larson, Renuka Sriram, Stephanie J. Blocker, Stephen Pickup, Alexandra Badea, Cristian T. Badea, Thomas E. Yankeelov, Thomas L. Chenevert

**Affiliations:** 1 Department of Cancer Systems Imaging, University of MD Anderson Cancer Center, 1881 E. Road, Houston, TX 77030, USA; 2 Department of Radiology, University of Washington, Seattle, WA 98105, USA; 3 Department of Radiology and Biomedical Imaging, University of California San Francisco, San Francisco, CA 94158, USA; 4 Department of Radiology, University of Michigan, Ann Arbor, MI 48108, USA; 5 Oden Institute for Computational Engineering and Sciences, and Livestrong Cancer Institutes, Dell Medical School, The University of Texas at Austin, Austin, TX 78712, USA; 6 Department of Radiology, Molecular Imaging Program at Stanford, Stanford University School of Medicine, Stanford, CA 94305, USA; 7 Mallinckrodt Institute of Radiology, Washington University, St. Louis, MO 63110, USA; 8 Fred Hutchinson Cancer Center, Seattle, WA 98109, USA; 9 Lester and Sue Smith Breast Center, Dan L Duncan Comprehensive Cancer Center, Houston, TX 77030, USA; 10 Center for In Vivo Microscopy, Department of Radiology, Duke University School of Medicine, Durham, NC 27710, USA; 11 Department of Radiology, University of Pennsylvania, Philadelphia, PA 19104, USA; 12 Department of Radiology, Duke University, Durham, NC 27708, USA; 13 Department of Biomedical Engineering, Diagnostic Medicine, and Oncology, Oden Institute for Computational Engineering and Sciences, Livestrong Cancer Institutes, The University of Texas at Austin, Austin, TX 78712, USA; 14 Department of Imaging Physics, MD Anderson Cancer Center, Houston, TX 77030, USA

**Keywords:** reproducibility, templates, quantitative imaging, consortia

## Abstract

Providing method descriptions that are more detailed than currently available in typical peer reviewed journals has been identified as an actionable area for improvement. In the biochemical and cell biology space, this need has been met through the creation of new journals focused on detailed protocols and materials sourcing. However, this format is not well suited for capturing instrument validation, detailed imaging protocols, and extensive statistical analysis. Furthermore, the need for additional information must be counterbalanced by the additional time burden placed upon researchers who may be already overtasked. To address these competing issues, this white paper describes protocol templates for positron emission tomography (PET), X-ray computed tomography (CT), and magnetic resonance imaging (MRI) that can be leveraged by the broad community of quantitative imaging experts to write and self-publish protocols in protocols.io. Similar to the Structured Transparent Accessible Reproducible (STAR) or Journal of Visualized Experiments (JoVE) articles, authors are encouraged to publish peer reviewed papers and then to submit more detailed experimental protocols using this template to the online resource. Such protocols should be easy to use, readily accessible, readily searchable, considered open access, enable community feedback, editable, and citable by the author.

## 1. Introduction

There has been a slowly unfolding crisis of confidence both in the scientific and lay community about the reproducibility and applicability of scientific research. A privately funded study of biomedical research applications identified deep challenges in reproducing key articles [1]. While some have suggested this is due to complicated physical experiments often produced in biomedical research, the reproducibility challenge was also found in digital data science research and psychological studies that do not require physical experimentation or exotic reagents [2,3]. These examples would seem to obviate the class of biomedical research as a special case. Amongst these studies, there is a common thread that the best predictor of reproduction was the active participation of the original research team or even the ability to reach the team members. This implies that there is a need for the space or ability to communicate all the necessary details within a traditional publication to enable reproduction by practitioners of the art. In the era of growing approved artificial intelligence (AI) applications to aid diagnostic image interpretation [4], there are continuing demands for robust curation and sustainable documentation of imaging data acquisitions and analyses. Quantitative imaging adds more reproducibility challenges related to assessment of confidence intervals or other statistical metrics for derived imaging biomarkers [5,6,7]. While quantitative standardization across sites may indeed prove difficult, protocols that effectively communicate methods may achieve reproducibility across sites without quantitative standardization. For example, capturing a color image of a slide is regularly communicated and achieved in the scientific and medical community without spectrally standardized light microscopes. Such standardization would indeed improve both communication of protocols and be, ultimately, reproducible, but quantitative standardization across sites is not absolutely required for the reproducibility of conclusions in biomedical research. Regardless, defining the required and desired limits of agreement between pre-clinical molecular imaging sites depends upon both the application and project goal and is outside of the scope of this white paper.

In broad readership journals with high impact factors, better named, citation factors, the entire imaging and post-image analysis protocols are typically only briefly summarized by a short paragraph in the methods section with, possibly, a figure. This is, likely, an insufficient space for detailed quantitative imaging analysis protocols and methods. For example, it is rare to publish instrument validation. An often unused but possible solution is the addition of detailed protocols and site validation in the online supplemental information section. Although imaging-specific journals may have a format with more space for methods and validation, the current academic incentives reward publication in broad-based journals with higher citation factors. Critically, there are no mechanisms in the literature, supplemental information or otherwise, to update or sunset protocols. These structural limitations are ultimately rooted in page costs for journals that maintain print publications. 

In the digital age, space constraints of the journal have effectively been lifted, and access to many bits of data are global and affordable. In fields such as immunology and cell biology, this has led to the development and publication of the Structured Transparent Accessible (STAR) protocols (Cell Press), which are much longer in-depth methods. Additionally, peer reviewed articles such as JoVE can include video content along with hyperlinks, text, and graphs. Indeed, the proliferation of multi-media methods reminds us that the history of science includes not only the written journal article and their associated methods, but also physical demonstrations to both convince the audience and to communicate discoveries. While multi-media methods are not a full substitute for a true “in person” demonstration, a detailed and multi-media method can reside online, and, therefore, are available for reference across many geographies and time zones. Additionally, the author can still be contacted for further clarification and potentially in person demonstrations. Several websites (e.g., protocols.io and github.io) can store digital protocols and are already utilized by the scientific community. Such digital, web-based platforms may be better suited for describing quantitative imaging methods than short methods in journal articles. Web-based platforms create the opportunities for general community input and adaptation of the protocols. Finally, online resources can additionally serve as a repository for individual investigators and bridge knowledge gaps during the natural ebb and flow of laboratory personnel.

Can building an online template and providing published protocols based upon those templates improve reproducibility? The RSNA Quantitative Imaging Biomarker Alliance (QIBA) process [8] has improved clinical quantitative imaging reproducibility [9,10,11,12] such that imaging can now be an endpoint at the FDA [13]. Certainly, pre-clinical research is not and should not be scoped and scaled the same as a validated process for determining the efficacy of a clinical drug. That caveat aside, the new policy at the FDA is a promising exemplar, since pre-clinical studies are a critical step in drug development, and animal model imaging is playing an increasing role as a corollary to non-invasive imaging in clinical trials and patient management applications. Finally, building a PIP might indeed have fewer ethical and legal challenges as the exemplar data and images will not consist of patients and patient images, but, rather, phantoms and pre-clinical models where anonymity is not an issue. Indeed, investigators should use a different process such as QIBA for communicating clinical protocols.

The authors reached a consensus that providing a template to produce highly detailed protocols could be leveraged by researchers to more effectively communicate their experiments and potentially improve reproducibility. Thus, the image-acquisition and data processing (IADP) working group of the NIH sponsored Co-Clinical Imaging Research Resource Program (CIRP) network sought to provide an easy-to-use template for more effective communication of pre-clinical imaging protocols, starting with pre-clinical PET, MRI, and CT. The IADP Working Group of the CIRP was tasked with establishing general guidelines and consensus in designing, optimizing, and applying best practices, for standardized operating procedures for preclinical quantitative imaging to support co-clinical trials across different imaging modalities including MRI, CT, PET, and, potentially, SPECT, represented by the various groups in CIRP. The goal is for the research community to leverage these templates to write and publish a pre-clinical imaging protocol or PIPs. Critically, a PIP should contain the whole experimental chain from the claim for achievable precision and accuracy through to the instrument validation and methods, and analyses must be presented and preserved to generate reproducible quantitative imaging measurements. These templates and protocols should be saved in a publicly searchable and addressable website. Secondarily, protocols could also serve as a starting point for defining acquisition settings that are similar to the biomarker presented. Readers and end users recognize and accept that they can and should modify these starting settings as needed for our/their own research as experts in their fields.

## 2. Methods

### 2.1. Identification of the Possible Use Cases

As a first step in developing a protocol template, likely use-cases for pre-clinical imaging protocols were enumerated. While there are many possible use cases for pre-clinical imaging protocols. The following use cases and examples were considered when developing the pre-clinical imaging protocol templates.

- Disseminating a new material/method combination: A new injectable reporter/imaging method was developed, and the authors want early adopters, particularly at other institutions or globally, to be more successful in their early pilot experiments. Thus, a new PIP was written and published.
- Documenting an imaging process for testing new drugs: A robust method was developed for proving target engagement for a class of drugs. A pre-clinical imaging protocol (PIP) was written, thus novel and emerging drugs in the same class can be tested for superior target engagement in vivo.
- Reporting new instrument tolerances: New tighter instrument tolerances such as resolution were developed that enable new applications. A PIP was written and uploaded for others to adjust tolerances.
- Empowering the community of researchers: Many research protocols have reasonable similarities but not identity, e.g., similar animal models; tumor locations; and assumed mechanisms of action such as cytotoxic treatment. A detailed PIP could be used by the research community to jumpstart their new investigations.
- Improving serial study designs: A community of non-imaging researchers have used invasive methods to assess therapeutic efficacy and have relied on group comparisons (e.g., treated vs. control groups). They hypothesized that greater sensitivity or specificity can be achieved through a non-invasive serial study. A published PIP provides a reasonable estimate of “precision” of a relevant QIB, which was useful in their serial study design. These estimates of precision were particularly helpful for order of magnitude power analysis (size of N).
- Accelerating clinical translation: It is anticipated that the inclusion of additional important details in PIPs (e.g., details of phantoms; analysis methods/stats; animal model, contrast agents) may enable translational researchers to more quickly and effectively design companion imaging trials.
- Providing a sustainable historical record: PIPs could provide detailed long-term records of the lab’s own investigations, which are useful to incoming and established members of the laboratory.

### 2.2. Development Process and Vetting

**Figure 1:**
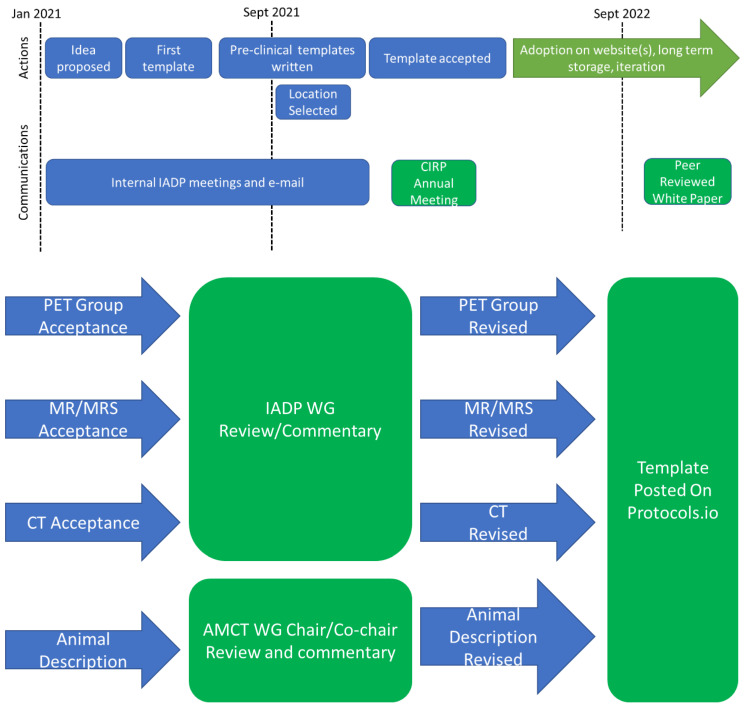
The timeline and workflow for development of Pre-clinical imaging workflows. (**Top**) The linear timeline from the proposal of the idea, drafting of the first template (MRI), and then extension and validation by other groups. (**Bottom**) The high-level workflow for production, review, and revision of pre-clinical imaging templates. Abbreviations: CIRP: Co-clinical Imaging Research Program; IADP: Image Acquisition and Data Processing; WG: working group; MRS: magnetic resonance spectroscopy; PET: positron emission tomography; CT: computerized tomography.

Draft templates were built for PET, MRI, and CT. Sub-committees were formed and comprised of experts from multiple institutions. Each committee held deep expertise in pre-clinical imaging and translation. Each modality sub-committee reviewed and provided feedback to the protocol template in an iterative process. The overall timeline and workflow are captured in Figure 1.

## 3. Developmental Boundaries and Constraints

### 3.1. Top Level Boundaries and Constraints

The PIPs were not designed to serve as a replacement for a publication, but, rather, as a means to provide more detailed methods section that were often not adequately described in traditional publication. Moreover, by following a standardized format, creation of new PIPs and PIP templates were, likely, simplified. Additional time and resources will not be allocated for writing or housing the protocols; however, original posting, amendments, and curation and storage of PIPs must be easy and nearly free.

### 3.2. Template Location Sustainability Requirements and Constraints

Given the above requirements, considerable thought and discussion was devoted to the site to house the templates. There were several key criteria for determining the location of the pre-clinical imaging protocol templates and pre-clinical imaging protocols. There were several absolute requirements: web accessibility, storage for both templates and protocols, author revision, date stamping, and protocol “sun setting”. Furthermore, there were a series of desired characteristics that were used to prioritize candidate sites: free accessibility, not-for-profit status of website owner, version tracking, and authorship tracking.

## 4. Results

Several sites were considered for housing protocol templates and protocols based upon the criteria put forth in Template Location Sustainability Requirements and Constraints. Table 1 annotates the reasoning of the IADP WG. Protocols.io was selected to house both the templates and the protocols. Given that github.io met all the key criteria as well, github.io can serve as a backup location for the PIP templates should there be a fundamental change to protocols.io.

**Figure 2:**
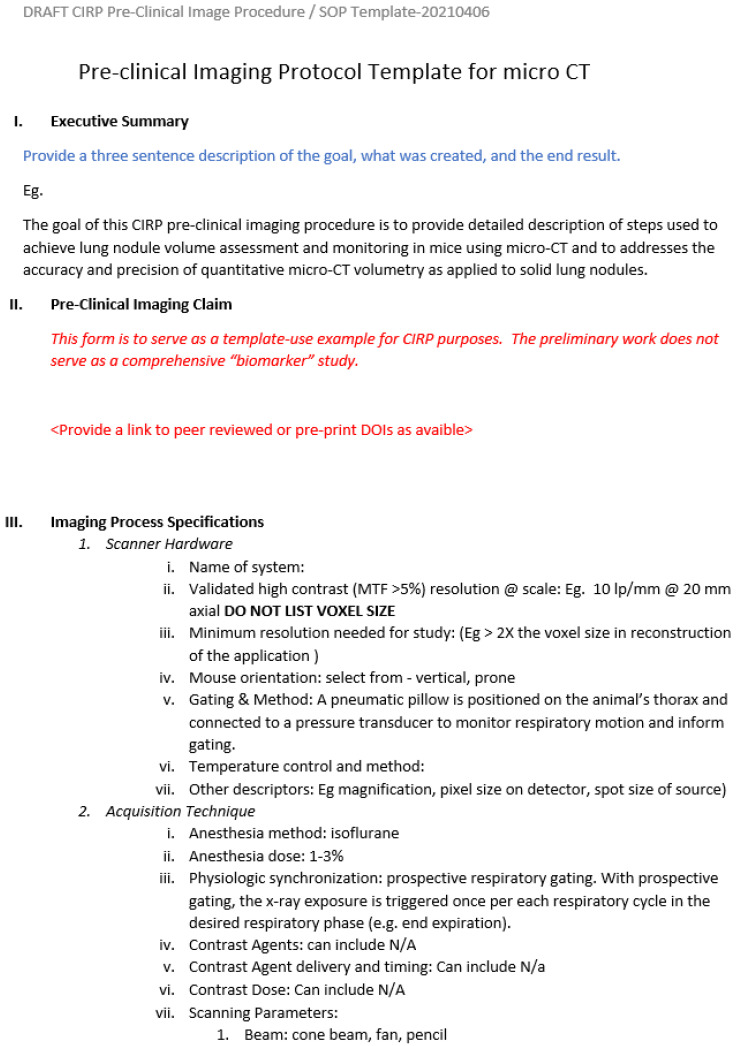
Screen capture of the first page of the pre-clinical imaging template for CT. Blue text indicates commentary, red text indicates instruction.

Protocol templates and instructions are posted on the protocols.io website. For each image modality, a protocol template was designed to capture critical modality specific information while still presenting a harmonized layout across modalities. Following the QIBA template, each protocol has an executive summary that provides a brief abstract to the protocol. Next, the claim for the biomarker precision and accuracy achieved for the pre-clinical imaging protocols are contained therein. This must include all parts of the protocol from instrument to contrast agents, animals, and analytics. The process of imaging acquisition is then described including validation of instrument performance. Then, there are sections for describing the animal model and quantitative metrics and associated data. The fields are free-write fields in a document. Protocol authors must minimally include searchable text and may include images or movies within the document (e.g., screen capture Figure 2). At each level, there are also descriptions of what information should be included, and, when possible, example values are provided for each element. As parameters evolve or change, there will be the ability to modify the template to include additional sub-attributes by bringing a request to the IADP sub-committee. Should the sub-committee expire, the duty could reside within the appropriately housed society expertise, such as the World Molecular Imaging Society (https://www.wmis.org/, accessed on 20 March 2023). The location of the protocol templates is https://www.protocols.io/workspaces/pre-clinical-imaging-protocols, accessed on 20 March 2023. These templates are ready for download and use by the research community. Researchers can then upload their own PIP into their own protocols.io folder. From once on protocols.io, PIPs can be found by other researchers through text search provided by the website.

A screen capture of the pre-clinical imaging protocol has been provided as an example of a protocol template. Full templates are intentionally not provided within this article to prevent version confusion. The approved templates are located at https://www.protocols.io/workspaces/pre-clinical-imaging-protocols (accessed on 20 March 2023). Inclusion of the templates within the article, while potentially easier to access for the initial readers, creates the opportunity for version mismatches when templates are updated and stored in the online repository.

## 5. Discussion

Low cost and ease of use were the two primary considerations during the designing protocol templates and the PIP process. First, the templates were hosted online for free. Any user can then, for free, download the template, fill out the template, and upload it to their own free protocols.io site. This enables researchers around the world to search protocols.io for the posted PIP and download a PIP for free. Because the templates and protocols were stored using web-based hosting, links could be disseminated on institutional web pages, personal web pages, society pages, or professional websites. Finally, protocol templates were designed to be facilitate re-purposing if multiple PIPs were created. For example, once the instrument validation section had been built for a PET or CT protocol, most of the instrument validation material could be copied over into the next protocol, unless there was a critical protocol specific quality assurance process.

There are several risks to broader adoption and long term utilization of templates and protocols. First, there is a concentration risk (the risk of a single vendor) by housing both protocols and templates on protocols.io, e.g., failure of the company or movement to a paywall. The group selected GitHub as an alternate to de-risk a movement to paywall at protocols.io. Second, there is the philosophical risk that pre-clinical imaging experiments are, ultimately, not reproducible due to some other driving root cause. One example might be the lack of quantitative agreement across pre-clinical imaging vendors. This is a possible contributor. However, the production of detailed protocols might indeed help highlight the need for quantitative agreement standards, if that is a primary driver of pre-clinical experimental reproducibility, and aid in the construction of such standards by the field. Such an effort was beyond the scope of this project.

Though the template and PIP process were created using QIBA “Profiles” as a model, it is important to acknowledge the necessary and appropriate differences in rate constants in the pre-clinical vs. clinical QIBA innovation cycle. Multiple peer-reviewed publications from multiple investigators describing consistent promising biomarker characteristics, ideally performed on multiple scanner platforms, that collectively meet adequate statistical rigor is a prerequisite for QIBA Profile’s development [8]. QIBA profile development, largely fueled by volunteer effort, typically takes several years to advance through multiple formal stages including public review consensus toward confirmation [9,10,11,12]. In clinical practice, once a protocol has been robustly demonstrated to positively impact clinical diagnosis and care, one desires to standardize that practice, to disseminate it broadly, and to impact public health. Due to evident medical risks in unacceptably rapid dissemination of untested/unproven technology, clinical adoption appropriately evolves over long periods of time. Therefore, the associated predictive imaging protocols persist and yield returns on time and effort investments over many years. The clinical research enterprise can support more expensive and expansive investment of time/money equivalences into the process of developing clinical protocols. Whereas, in pre-clinical research, medical risk is low, and innovation rate is high. Thus, for rapid adoption, the PIP templates and posting process must be easy to use, cost little money or time, and provide immediate benefit to the researching end user. While a significant effort was expended to achieve these goals, time and utility to the community will, ultimately, govern the net adoption of the templates and protocols.

This effort described herein is harmonized with other projects within the CIRP network. Through developing an automated capture of prioritized metadata in a pre-clinical DICOM format, many of the data fields found in the template can be referenced and inserted by the author of a pre-clinical imaging protocol. The templates described herein were designed to enable the CIRP network members to publicly disseminate protocols developed by their groups as mandated by CIRP funding mechanism. Indeed, as of the writing of this article, several are already published and public on protocols.io. Finally, PIPs could help enable the dissemination of scientific data as required by the new NIH funding directive (https://sharing.nih.gov/data-management-and-sharing-policy/, accessed on 20 March 2023).

There are several envisioned future directions. First early adopters will be able to provide feedback on the protocols.io website. This feedback can be later incorporated into subsequent editions of the template via the IADP working group. Second, new PIP templates could be developed for: magnetic resonance spectroscopy (MRS), hyperpolarized MRI, single photon emission computed tomography (SPECT), optical, and emerging techniques. Importantly, the pre-clinical imaging community is a global community. Language localization of templates and protocols can be achieved through collaboration with global molecular imaging societies. These societies include, but are not limited to, the South American, European, Asian, and Middle Eastern molecular imaging societies. Finally, a meta-analysis of PIPs published in protocols.io might be leveraged to build a public database of protocols with a novel and easy-to-use graphical user interface.

## Figures and Tables

**Table 1:** Key discussion points and actions around locations to house pre-clinical protocols and templates.

Suggested Hosting Location	Advantages
MICAD	Website already exists
CIRP HUB	Website already exists
NCI hosting	Could be publicly accessible and built for purpose
Journals Articles—SI or appendix	System exists, protocols are publicly accessible, funding included in page charges
STAR protocols & manuscripts	Website: already exists, is publicly accessible, used in biology
Github	Website: already exists, publicly accessible, non-for profit, free
Protocols.io	Website: already exists, publicly accessible, non-for profit, free, can reference PLOS one, leveraged by PDX consortium
Suggested Hosting Location	Challenges
MICAD	No funding, time, or personnel to update site
CIRP HUB	Website is small and not publicly accessible
NCI hosting	No funding, time, or personnel to create site
Journals Articles—SI or appendix	Cannot update or sunset protocols, template adoption may be difficult
STAR protocols & manuscripts	Cannot update or sunset protocols, template adoption may be difficult, need journal to adopt templates and template updates
Github	Solution primarily used in software development, may need to adapt format for biological protocols
Protocols.io	Currently free but may be moving to paid subscription model
Suggested Hosting Location	Consensus Action
MICAD	Not selected
CIRP HUB	Not selected
NCI hosting	Not selected
Journals Articles—SI or appendix	Not selected
STAR protocols & manuscripts	Not selected
Github	Selected as backup
Protocols.io	Selected as preferred site

## Data Availability

There is no data in this white paper. The protocol templates are freely available to the public via protocols.io as indicated above.
